# X-Ray Weld Image Detection Method of Water Injection Network Based on Sparse Representation

**DOI:** 10.3390/s26134160

**Published:** 2026-07-01

**Authors:** Hailong Liu, Weixin Gao, Li Gao, Junjie He

**Affiliations:** 1 School of Electronic Engineering, Xi’an Shiyou University, Xi’an 710065, China; liuhailong@xsyu.edu.cn (H.L.); wxgao@xsyu.edu.cn (W.G.); qdliukk@126.com (J.H.); 2 Key Laboratory of Exploration and Development of Complex and Difficult-to-Produce Oil & Gas Reservoirs (Xi’an Shiyou University), Ministry of Education, Xi’an 710065, China

**Keywords:** water injection network, X-ray weld image, sparse representation, defect detection

## Abstract

**Highlights:**

**What are the main findings?**

- A sparse representation-based framework is proposed to detect micro-defects (e.g., cracks and pinholes) in X-ray weld images of water injection networks. By combining Otsu thresholding with Sobel edge detection, segmented ROI extraction is achieved, which adapts to inclined or curved welds and effectively reduces background interference.
- Using dictionary learning (K-SVD, OMP) and a unified 15 × 15 template for defect SDR normalization, the method achieves 99.81% overall recognition accuracy for circular defects, linear defects, and noise samples, demonstrating strong capability in identifying low-contrast, small-sized defects.

**What are the implications of the main findings?**

- The proposed method provides an accurate and robust automatic solution for detecting micro-defects in X-ray weld images of water injection networks, advancing intelligent nondestructive testing for pipeline welds.
- For larger defects such as lack of fusion, incomplete penetration, and cracks, the sparse description approach still exhibits missed detections. Therefore, integrating it with a YOLO-based deep learning model is recommended to achieve comprehensive detection of all weld defect types.

**Abstract:**

X-ray testing is a cornerstone nondestructive testing (NDT) technique in the nondestructive testing of welds. To address the challenges posed by minute defects such as cracks and pinholes—characterized by small size, weak features, and a tendency to be confused with noise—this paper proposes a minute defect recognition framework based on sparse representation. (1) Median filtering was selected as the basic denoising method. In combination with image enhancement, the discriminability of weld regions and defect features was improved. (2) A segmented ROI extraction method combining Otsu threshold segmentation and Sobel edge detection was proposed. This method can better adapt to inclined or curved weld images and effectively reduce background interference. (3) A micro-defect recognition method based on sparse representation was proposed. By constructing an SDR and combining dictionary learning with sparse solving models, effective representation and classification of micro-defect regions were achieved. Its effectiveness and engineering application value were verified through actual engineering data, third-party witness tests, and competition results.

## 1. Introduction

Waterflooding has become the predominant development technique for domestic oilfields in China. The X-ray weld images of water injection system pipelines typically exhibit characteristics such as low contrast, high noise, complex backgrounds, and small defect targets. When conventional methods are employed for recognition, difficulties in feature extraction and insufficient detection accuracy often arise [1]. Existing weld defect recognition methods can be mainly categorized into several types: those based on object detection frameworks, those based on deep learning feature representation, and those based on fusion techniques [2].

While deep learning methods have achieved significant success, they often require large-scale datasets and substantial computational resources [3]. Methods based on object detection frameworks, such as DSF-YOLO, proposed by Zhang et al. [4], integrates dynamic staged feature fusion to address blurred boundaries. Similarly, Su et al. [5] and Liang et al. [6] have improved detection accuracy using attention mechanisms and real-time detection strategies, respectively. On the other hand, methods based on deep feature representation, such as those utilizing ResNet50 [7] or improved VGG architectures [8], focus on enhancing classification precision. However, these data-driven models may struggle when applied to micro-defect detection where sample scarcity and low contrast are prevalent.

In contrast to the “black-box” nature of deep learning, sparse representation theory offers a more interpretable mathematical framework for signal reconstruction. It holds that an image signal can be represented as a linear combination of a small number of basis elements (atoms) from an overcomplete dictionary. This approach has demonstrated advantages in feature expression and anti-noise interference, particularly suitable for signals with distinct local structures.

In the context of nondestructive testing, sparse coding has been explored for fault diagnosis and image denoising. For instance, Aharon et al. [9] proposed the K-SVD algorithm, which has become a cornerstone for designing overcomplete dictionaries. Mairal et al. [10] further advanced online dictionary learning, demonstrating its effectiveness in image restoration. However, when applied to micro-defect detection in radiographic images, existing methods often struggle with the high coherence among atoms [11] and the difficulty in distinguishing weak defect features from strong background noise [12]. While Zhang et al. [13] introduced discriminative dictionary learning for pattern recognition, its adaptation to the specific characteristics of weld micro-defects (e.g., pinholes and micro-cracks) remains underexplored. However, existing sparse representation methods for weld defects often face challenges in two aspects: (1) The dictionary learning process may result in high coherence among atoms, leading to redundancy and weakened discriminative power. (2) The sparse solving strategy for micro-defects (e.g., micro-cracks and pinholes) is often sensitive to background interference, especially when the defect size is extremely small relative to the Region of Interest (ROI) [14,15,16].

To address the challenges posed by minute defects in X-ray weld images—characterized by small size, weak features, and a tendency to be confused with noise—this paper proposes a micro-defect recognition framework based on an improved sparse representation. Unlike generic deep learning approaches that treat defects as detection targets, or standard sparse models that use global features, the scientific novelty of this work lies in the following aspects:

Methodological Level: We propose a segmented ROI extraction method combining Otsu thresholding and Sobel edge detection, specifically designed to adapt to inclined or curved welds, thereby reducing background interference for subsequent sparse coding.

Algorithmic Level: We introduce a density-based clustering algorithm to screen candidate regions, constructing Sparse Defect Regions (SDRs) as input units. Furthermore, we optimize the dictionary learning model by incorporating orthogonal constraints and expected projection minimization to reduce atom correlation.

Engineering Application: Through rigorous experimentation, we determine a unified 15 × 15 template size for SDR normalization, which balances computational efficiency and feature representation capability. The method achieves 99.81% overall recognition accuracy for circular defects, linear defects, and noise samples, demonstrating strong capability in identifying low-contrast, small-sized defects.

The remainder of this paper is organized as follows: Section 2 details the image preprocessing and ROI extraction methodology. Section 3 elaborates on the sparse description model, including dictionary construction and sparse solving. Section 4 presents the experimental validation, and Section 5 concludes the paper.

## 2. Method for X-Ray Weld Image Preprocessing and Evaluation Region Extraction

**Figure 1:**
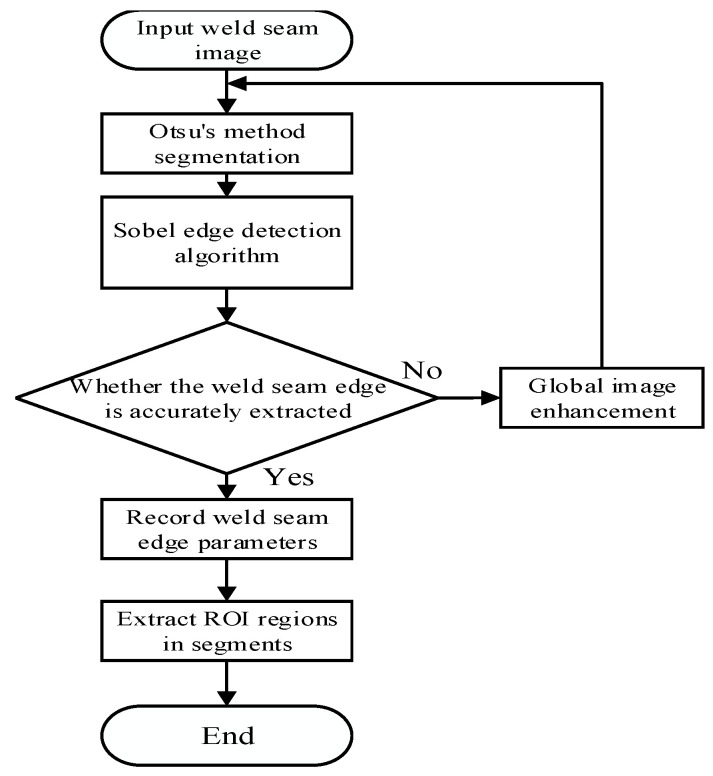
Flowchart of segmented extraction of weld ROI (Region of Interest).

As described above, automated localization and segmented extraction of the evaluation region to highlight the key information of the weld and the heat-affected zone constitute a prerequisite for accurate defect detection and welding quality assessment. The specific procedure is illustrated in Figure 1.

**Figure 2:**
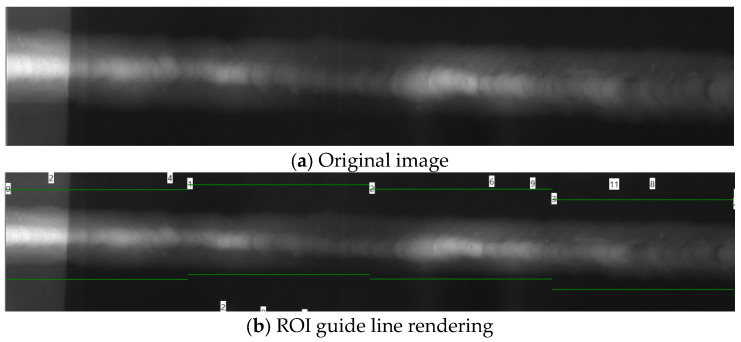
Schematic diagram of ROI guide lines for weld seam segmentation.

This method processes the image in segments along the weld direction, enabling each segmented ROI to closely conform to the boundary morphology of the weld. As a result, background interference is reduced and the accuracy of weld region extraction is improved. Moreover, the segmentation approach allows for local precise cropping of the weld region, thereby enhancing the stability of defect detection. The effect of generating the guided lines for the segmented ROIs is shown in Figure 2. The numbers in the figure denote the segment indices for the piecewise linearization of the ROI. The length of the green line corresponds to the length of one linearized segment.

## 3. Sparse Description-Based Method for Small Defect Recognition

Existing weld defect recognition algorithms still exhibit differences in feature representation capability, small-sample adaptability, interpretability, and computational complexity when addressing the challenges of small-diameter pipe X-ray weld images, such as the small size of tiny defects, weak local features, strong background interference, and limited sample availability, as shown in Table 1.

Compared with traditional image processing methods, statistical learning methods, and deep learning methods, the sparse description approach constructs a representative dictionary and represents the test sample as a linear combination of a few atoms, thereby effectively characterizing the local structural features of tiny defects.

### 3.1. Basic Idea of Sparse Description

The basic idea of sparse description is that a test sample can typically be linearly represented by a small number of representative atoms from a dictionary. Let the test sample be $y\in R^{n}$, the dictionary matrix be $D=[d_{1},d_{2},\cdots ,d_{K}]\in R^{n\times K}$, and $d_{i}$ be the i-th atom in the dictionary. Then the sample y can be expressed as

(1) y=Dα

where $\alpha =[\alpha_{1},\alpha_{2},\cdots ,\alpha_{K}{]}^{T}$ is the representation coefficient of the sample over the dictionary. When only a few elements in α are non-zero or approximately non-zero, the sample y is said to have a sparse representation over the dictionary D.

For images of tiny weld defects, different defect categories exhibit differences in local grayscale distribution, edge structure, and morphological features. Therefore, by constructing a dictionary that contains samples from different categories, the sparse coefficient distribution of a test sample over the dictionary can reflect its category attributes. If a sample matches well with a certain defect category dictionary, the coefficient responses of the corresponding atoms are typically more prominent, thereby providing a basis for subsequent defect recognition. Under ideal conditions, the sparse representation can be expressed as

(2) $$\min\|\alpha {\|}_{0}\;s.t.y=D\alpha$$

**Figure 3:**
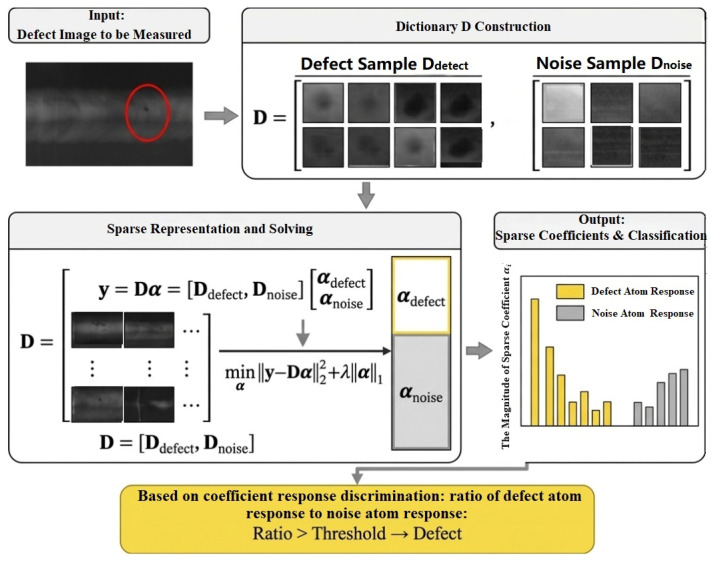
Principle of defect recognition based on sparse description.

Considering the presence of noise and reconstruction errors in actual images, it is typically written as

(3) $$\min\|\alpha {\|}_{0}\;s.t.\;\|y-D\alpha {\|}_{2}\leq \varepsilon$$

where $\|\alpha {\|}_{0}$ denotes the number of non-zero elements in the representation coefficient vector, and ε is the allowable reconstruction error threshold. Figure 3 shows a schematic diagram of defect recognition based on sparse description.

As shown in Figure 3, the key to sparse description lies in dictionary construction and sparse coefficient solving, and the dictionary learning and sparse solving algorithms need to be designed accordingly.

### 3.2. Basic Model of Sparse Description

**Figure 4:**
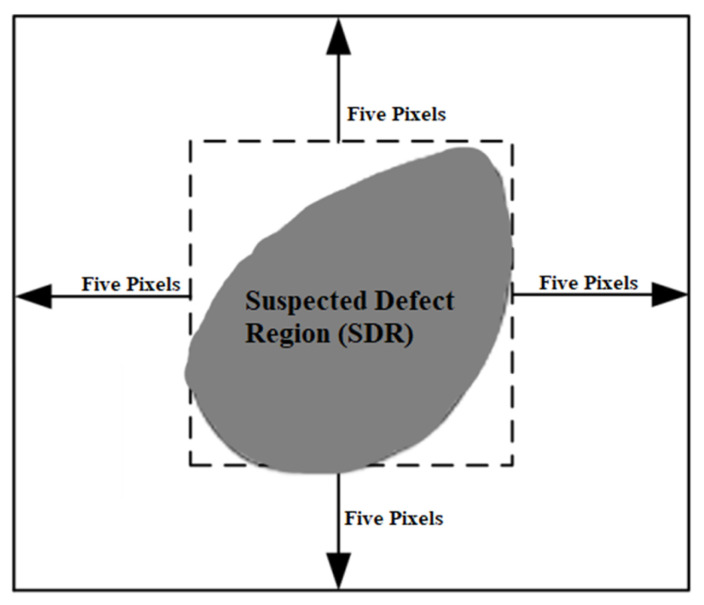
Schematic diagram of the SDR region definition.

To enable the sparse description model to characterize the features of tiny weld defects more effectively, it is further necessary to extract local samples that may contain anomaly information from the weld region. For weld images, the background is relatively complex, and tiny defects are typically small with varied morphologies. If sparse representation modeling is applied directly to the entire weld ROI region, a significant amount of irrelevant background information may be introduced, thereby affecting subsequent dictionary construction and classification performance. Based on the extracted weld ROI, this paper further constructs SDR (Sparse Defect Region) samples, which serve as the input for subsequent sample normalization, dictionary learning, and defect recognition. Figure 4 presents a schematic diagram of the SDR region definition.

**Figure 5:**
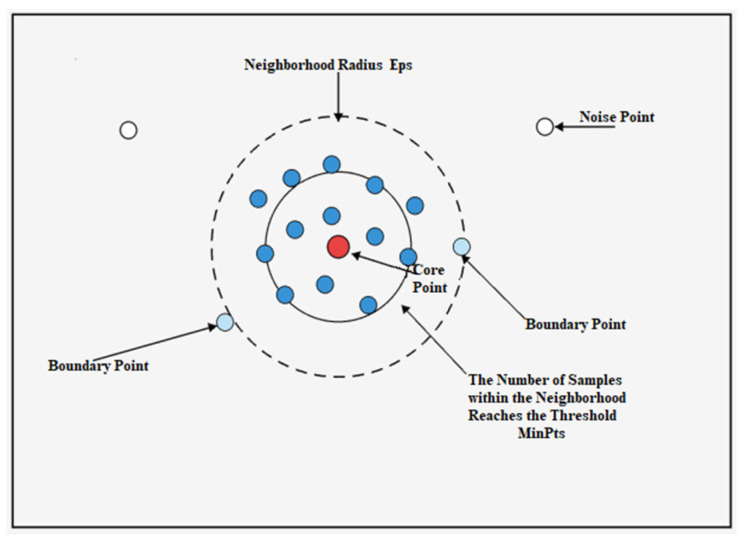
Schematic diagram of the density-based clustering algorithm.

To improve the accuracy of suspicious defect region extraction, this paper adopts a density-based clustering algorithm to screen candidate regions within the weld ROI. This method identifies clusters based on local density maxima, effectively distinguishing aggregated defects from isolated noise. Since actual defect regions in weld images typically exhibit a certain degree of local aggregation, whereas random noise is mostly discretely distributed, the density-based clustering approach is suitable for SDR sample extraction. Figure 5 shows a schematic diagram of the density-based clustering algorithm.

The procedure of segmenting SDR using the density-based clustering algorithm is as follows:

(1) Taking the extracted weld ROI as input, preliminarily locate candidate anomaly regions in the image.
(2) Extract features such as position, area, grayscale, and morphology of the candidate regions, and construct feature vectors.
(3) Set the neighborhood radius Eps and the minimum number of samples MinPts for the density-based clustering algorithm.
(4) Perform cluster analysis on the candidate regions in the feature space, grouping densely distributed samples into the same cluster.
(5) Identify isolated and scattered regions that do not form valid clusters as noise and remove them.
(6) Retain the valid candidate regions obtained after clustering as the SDR extraction result.

**Figure 6:**
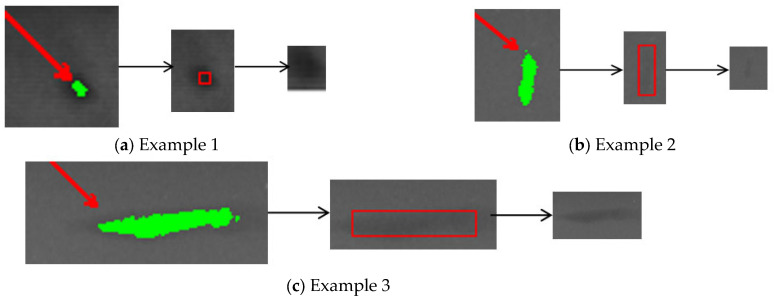
Segmentation map of the density-based clustering algorithm.

An example of an SDR extracted by the density-based clustering algorithm is shown in Figure 6. Through the above processing, background interference can be reduced while preserving the local key information of defects, thereby providing more effective input samples for subsequent sparse description modeling.

### 3.3. Dictionary Matrix Problem Description

To facilitate a unified representation of weld defect samples, the image samples first need to be converted into vector form. Let the size of a single normalized sample be m×n. Then it can be expanded into an *mn*-dimensional column vector, denoted as $x_{i}\in R^{mn}$. For multiple samples of the same category, arranging them sequentially in columns constitutes the corresponding sample matrix.

For the problem of tiny weld defect recognition, candidate regions may contain either actual defects or noise/pseudo-defect regions, and different SDR samples often share certain similarities. Therefore, dictionary construction should take into account not only the representational power of the samples but also the representativeness of the atoms and their class-discriminative capability.

Let the training sample set be *Y*, the dictionary matrix be *D*, and the sparse coefficient matrix corresponding to the samples be *A*. Then the basic objective of dictionary learning can be expressed as

(4) $$\underset{D,A}{\min}\|Y-DA{\|}_{F}^{2},\;s.t.\;\|\alpha_{i}{\|}_{0}\leq T$$

where $\|\cdot {\|}_{F}$ denote the Frobenius Norm, *α_i_* is the sparse coefficient vector corresponding to the *i*-*th* training sample, and *T* is the sparsity constraint.

Considering the multi-class characteristics of weld defects, different classes of SDR samples exhibit differences in local grayscale distribution, edge structure, and morphological features. Therefore, separate training sample subsets are constructed according to each class. Let the set of samples belonging to class cc be *Y*^(*c*)^, and its corresponding sub-dictionary be *D*^(*c*)^. Then the learning model for the class-specific sub-dictionary can be expressed as

(5) $$\underset{D^{\left(c\right)},A^{\left(c\right)}}{\min}\|Y^{\left(c\right)}-D^{\left(c\right)}A^{\left(c\right)}{\|}_{F}^{2},\;s.t.\;\|\alpha_{i}^{\left(c\right)}{\|}_{0}\leq T,c=1,2,\cdots ,C\;$$

where *C* denotes the number of classes. Furthermore, by concatenating the sub-dictionaries of all categories, a complete dictionary matrix can be constructed as follows:

(6) $$D=\left[D^{\left(1\right)},D^{\left(2\right)},\cdots ,D^{\left(C\right)}\right]$$

**Figure 7:**
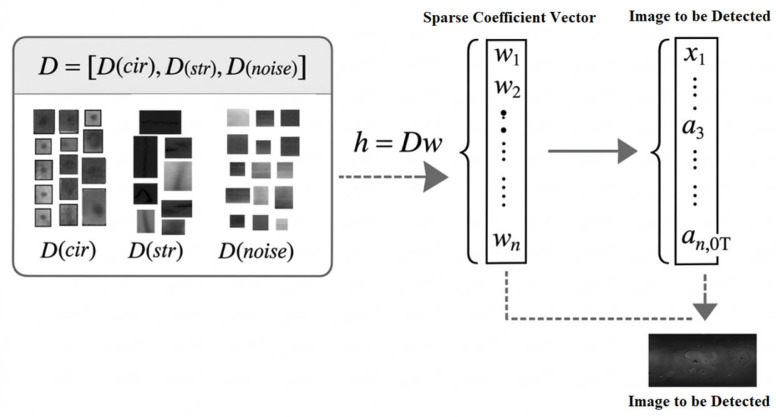
Schematic diagram of weld defect dictionary construction.

In actual weld images, in addition to real defects, the candidate regions may also contain noise samples or pseudo-defect regions. Therefore, noise samples should be appropriately incorporated to enhance the dictionary’s adaptability and discriminative capability for complex weld images. Furthermore, to reduce dictionary redundancy and improve its discriminative power, constraint terms need to be added to the dictionary learning model. The optimized formulation can be written as

(7) $$\underset{D}{\min}\left\{\left\|Y-DA{\|}_{F}^{2}+\lambda \;\Phi (D\right)\right\}$$

where *Φ*(*D*) denotes the regularization term used to constrain the coherence of dictionary atoms, and λ is a balance parameter. This model, while controlling the sample reconstruction error, can further enhance the discriminability of dictionary atoms. Figure 7 present a schematic diagram of the weld defect dictionary construction.

### 3.4. Algorithm for Constructing Dictionary Learning Model

From the dictionary learning model, both the dictionary matrix and the sparse coefficient matrix are quantities to be solved, making it difficult to obtain an analytical solution directly. Therefore, an alternating iterative optimization approach is adopted: the sparse coefficients are solved with the dictionary matrix fixed, and the dictionary atoms are updated with the sparse coefficient matrix fixed. Commonly used learned dictionary construction methods mainly include the Method of Optimal Directions (MOD) and the K-SVD algorithm. A comparison of their characteristics is shown in Table 2.

**Figure 8:**
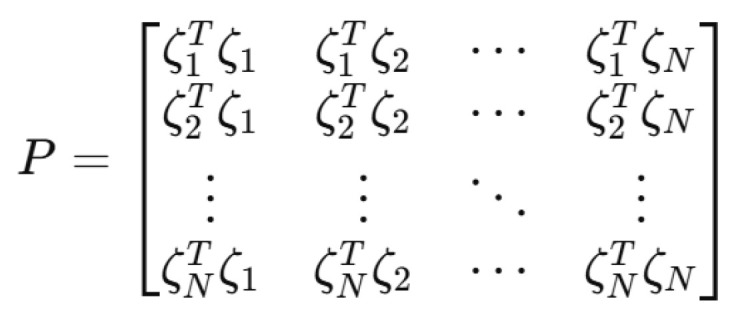
Schematic diagram of vector inner product relationships among training sample atoms.

Although the K-SVD algorithm can learn dictionary atoms with strong representational capability from training samples, due to the certain similarities in local structures among tiny weld defect samples, some of the learned atoms may still exhibit strong correlations. High redundancy among dictionary atoms not only increases the computational complexity of sparse solving but also weakens the discriminative capability among different classes of samples in the sparse representation process. Therefore, based on the learned dictionary, it is necessary to further investigate dictionary construction methods incorporating orthogonal constraints to enhance the independence and discriminative power of dictionary atoms. Based on the above idea, this paper formulates the dictionary optimization problem under the expectation projection constraint as

(8) $$\underset{\zeta^{N},A}{\min}\sum_{i=1}^{N}\sum_{j=i+1}^{N}\left|\zeta_{i}^{T}\zeta_{j}\right|,\;s.t.\;\|Z-\zeta^{N}A{\|}_{F}^{2}\leq \eta ,\|A{\|}_{F}\leq \gamma$$

where *N* denotes the number of atoms in the dictionary matrix, *ζ_i_* denotes the *i*-th dictionary atom, *Z* denotes the training sample matrix, *A* is the coefficient matrix corresponding to the samples, *η* is the reconstruction error threshold, and *γ* is the sparsity control parameter. The objective function reduces the correlation among atoms by minimizing the inner product between dictionary atoms, while the constraints limit the overall sample reconstruction error and the magnitude of the coefficient matrix, respectively, thereby ensuring that the selected dictionary maintains good sample representational capability while having low redundancy. Suppose the dictionary contains N column vectors $\zeta_{1},\zeta_{2},\cdots ,\zeta_{N}$. The vector inner product between any two atoms is denoted as $P_{ij}=\zeta_{i}^{T}\zeta_{j}$. To intuitively characterize the correlation relationships among dictionary atoms, a vector inner product correlation matrix of the dictionary atoms can be constructed, as illustrated schematically in Figure 8.

**Figure 9:**
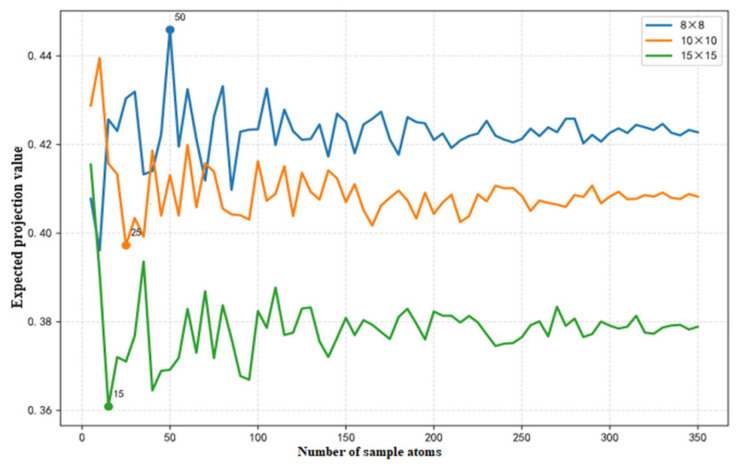
Expected curves of defect SDR for different template sizes.

**Figure 10:**
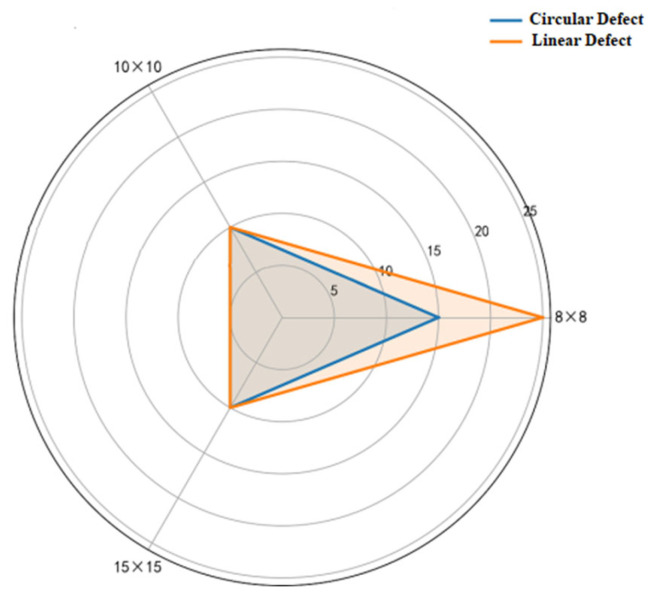
Comparison of optimal sample numbers for different templates.

To investigate the influence of different normalization template sizes on the construction of the defect SDR dictionary, this paper selects circular defect SDR and linear defect SDR samples for experimentation, and normalizes them to three template sizes: 8 × 8, 10 × 10, and 15 × 15. On this basis, the expected projection values under different numbers of sample atoms are calculated according to Equation (8), yielding the expected curves of defect SDR for different templates as shown in Figure 9. Meanwhile, the optimal numbers of samples for circular defect SDR and linear defect SDR under different templates are counted, and the results are presented in Figure 10.

As shown in Figure 9, with the increase in the number of sample atoms, the expected projection curves for the three template sizes gradually level off, indicating that the dictionary’s capability to characterize defect features is continuously enhanced. When the number of atoms reaches a certain range, further increasing the number of atoms no longer significantly improves the expected projection value. According to the inflection points of the curves, the corresponding atom numbers for the 8 × 8, 10 × 10, and 15 × 15 templates are approximately 50, 25, and 15, respectively, indicating that the 15 × 15 template achieves relatively stable defect characterization with a smaller number of atoms.

As shown in Figure 10, under the 8 × 8 template, the optimal numbers of samples for circular defect SDR and linear defect SDR are 15 and 25, respectively, whereas under the 10 × 10 and 15 × 15 templates, the optimal number of samples for both defect types are 10. In comparison, the 15 × 15 template requires a lower number of atoms while maintaining representational capability and exhibits better stability. Therefore, this paper ultimately selects the 15 × 15 template as the unified template size for defect SDR.

To determine the optimal scale of the dictionary matrix, we conducted experiments analyzing the relationship between the number of atoms and the expected projection value (as shown in Figure 9). The results indicate a clear “diminishing returns” phenomenon. Specifically, for the selected 15 × 15 template, the expected projection curve stabilizes when the number of atoms reaches approximately 15. Increasing the atom count beyond this point (e.g., to 25 or 50) does not significantly improve the expected projection value but would significantly increase the computational complexity of the OMP algorithm O(K mN). Therefore, based on the inflection point analysis in Figure 9 and the optimal sample count statistics in Figure 10, we determined that 15 atoms are the optimal setting for the 15 × 15 dictionary. This setting ensures sufficient representational power while maintaining high computational efficiency.

### 3.5. Sparse Solving and Classification Decision

After the dictionary construction is completed, it is necessary to solve the sparse representation coefficients of the test sample over the dictionary. Considering that the Orthogonal Matching Pursuit (OMP) algorithm features simplicity in implementation, high solving efficiency, and suitability for small-sample scenarios, this paper adopts a sparse solving method based on the orthogonal matching idea to obtain the sparse representation of the test sample.

Let the test sample be *y*, and the dictionary matrix be $D=[d_{1},d_{2},\cdots ,d_{K}]$. Let the sparse coefficient vector be α; then the sparse solving problem can be expressed as

(9) $$\min\|\alpha {\|}_{0}\;s.t.\;\|y-D\alpha {\|}_{2}\leq \varepsilon$$

where ε is the allowable reconstruction error threshold. The OMP algorithm gradually approximates the sparse solution of the sample by iteratively selecting the dictionary atom most correlated with the current residual and performing orthogonal projection onto the subspace spanned by the selected atoms. Its basic steps are as follows.

(1) Initialization

Initialize the residual vector, the support set, and the iteration counter.

(10) $$\begin{array}{c} \;r_{0}=y,\;\Lambda_{0}=\emptyset ,\;t=0\; \end{array}$$

where *r*_0_ is the initial residual and Λ_0_ is the initial support set.

(2) Select the Most Relevant Atom

At the t-th iteration, compute the correlation between the current residual and each dictionary atom, and select the atom index with the maximum correlation, i.e.,

(11) $$\lambda_{t}=\arg\;\underset{j}{\max}\left|d_{j}^{T}r_{t-1}\right|$$

(3) Update the support set

Add the index *λt* to the support set, and update the current support set as

(12) $$\Lambda_{t}=\Lambda_{t-1}\cup \left\{\lambda_{t}\right\}$$

(4) Least squares solution for coefficients

For the sub-dictionary $D_{\Lambda_{t}}$ corresponding to the current support set, the sparse coefficients are solved using the least squares method, i.e.,

(13) $$\alpha_{t}=\arg\;\underset{\alpha }{\min}\|y-D_{\Lambda_{t}}\alpha {\|}_{2}^{2}$$

(5) Update the residual

Update the residual vector according to the current sparse coefficients:

(14) $$r_{t}=y-D_{\Lambda_{t}}\alpha_{t}$$

(6) Termination Condition

If the residual satisfies the termination condition $\|r_{t}{\|}_{2}\leq \varepsilon$, or when the preset maximum sparsity is reached, the iteration terminates. Otherwise, set *t* = *t* + 1 and return to step (2) to continue the iteration. Through the above process, the sparse representation coefficients of the test sample over the dictionary can be obtained. For the task of weld defect recognition, the test sample typically exhibits a stronger response on the sub-dictionary corresponding to its own category; therefore, classification can be performed based on the distribution of sparse coefficients among the sub-dictionaries of different categories. Let the set of atom indices corresponding to the sub-dictionary of class cc be Λc; then the class of the sample can be expressed as

(15) $$label(y)=\arg\;\underset{c}{\max}\sum_{j\in \Lambda_{c}}|\alpha_{j}|$$

where *α_j_* denotes the *j*-*th* element of the sparse coefficient vector. The equation indicates that when the contribution of the sparse coefficients of the test sample on a certain class sub-dictionary is more significant, it can be classified as a defect of that class.

The computational complexity of the proposed sparse representation framework is primarily determined by the Orthogonal Matching Pursuit (OMP) algorithm during the testing phase. Let m denote the dimension of the sample vector (m = 15 × 15 = 225 in our case), N denote the number of atoms in the dictionary, and K denote the sparsity level (number of iterations).

The dominant operation in each OMP iteration is the computation of the inner product between the residual and all dictionary atoms, which has a complexity of O(mN). Since this process is repeated for K iterations, the overall computational complexity of the OMP algorithm is approximately O(K·m·N).

In our experiment, with a fixed sparsity constraint K and a dictionary size N (optimized to a relatively small scale as discussed in Section 3.4), the inference time per sample is significantly lower compared to the convolution operations required in Deep Neural Networks (DNNs) for full-image processing. This theoretical analysis suggests that our method offers a favorable trade-off between accuracy and speed, particularly suitable for scenarios requiring real-time or resource-constrained processing.

### 3.6. Dual-Thread Detection Mechanism for Comprehensive Defect Recognition

While the sparse representation method demonstrates superior performance in detecting micro-flaws, the detection of large-size weld defects (such as incomplete penetration and lack of fusion) presents a distinct challenge. In our research, we found that a single algorithm is insufficient to cover the vast morphological differences between micro- and macro-defects.

To address this, we have developed a Dual-thread Detection Mechanism:

Thread 1 (Micro-defects): Utilizes the sparse representation algorithm described in Section 4.1. This thread is optimized for low-contrast, small-area defects that are easily confused with background noise.

Thread 2 (Macro-defects): Utilizes a YOLO-based (You Only Look Once) algorithm specifically tailored for large-size defects.

Rationale for Separation: Large-size defects often exhibit complex textures and occupy a significant portion of the Region of Interest (ROI). The sparse representation model, which relies on linear combinations of atoms, can become computationally inefficient and less accurate when dealing with such large-scale variations. Conversely, the YOLO algorithm, with its global receptive field and deep convolutional features, is highly efficient at locating and classifying these extensive structural anomalies.

Technical Implementation: The system first segments the ROI using the hybrid strategy (Otsu + Sobel). The extracted ROI is then fed into both threads in parallel. If the sparse representation thread identifies a high-confidence micro-defect, it is flagged. Simultaneously, the YOLO thread scans for large defects. The final output is a fusion of results from both threads, ensuring no defect size is overlooked.

Reference to Detailed Methodology:

The detailed architecture, hyperparameter settings, and training protocol for the YOLO-based detection model have been thoroughly documented in our previous work [17]. In that study, we validated the model’s effectiveness on a similar dataset of pipeline welds. In this paper, we adopt that proven YOLO framework as the second thread to complement the sparse representation method.

## 4. Experimental Results and Analysis

### 4.1. Experiment on Sample Training of the Dictionary Matrix

To validate the effectiveness of the proposed sparse description-based weld defect recognition method, a weld image database containing several typical defect types was constructed. This dataset originates from actual engineering projects, specifically collected by technicians from the PipeChina (China Petroleum Pipeline Bureau) at construction sites across Northwest, Southwest, and East China.

The acquisition and construction process of the samples is as follows: Technicians first conducted preliminary screening on the original radiographic images. Suspected defective images were then sent to technical engineers who manually annotated the position and size of defects on digital images. Subsequently, a third-party inspection company re-examined and re-annotated these images according to Chinese national standards GB/T 44046-2024 [18], NB/T 47013-2015 [19], and GB/T 3323-2019 [20]. Finally, an expert panel meeting was held where multiple experts confirmed on-site whether the defects existed, whether the annotated sizes were correct, and whether the defect types were appropriate. After forming a unified opinion, the finalized dataset was returned to PipeChina.

The database includes 1000 circular defect samples, 500 linear defect samples, and 3500 noise samples, totaling 5000 sample images During the experiment, without setting any prior conditions, the proposed algorithm was directly applied to classify the defect types of the samples, and the experimental confusion matrix was recorded to analyze the discrepancies between the recognition results and the true categories.

To objectively evaluate the model performance, accuracy (ACC), precision (PPV), true positive rate (TPR), and true negative rate (TNR) are adopted as evaluation metrics, and their calculation formulas are as follows:

(16) $$\left\{\begin{array}{ll} ACC=\frac{TP+TN}{TP+TN+FP+FN}TPR=\frac{TP}{TP+FN} & \\ PPV=\frac{TP}{TP+FP}TNR=\frac{TN}{TN+FP} & \end{array}\right.$$

where TP (true positive) denotes the number of positive samples correctly identified as defects, TN (true negative) denotes the number of negative samples correctly identified as non-defects, FP (false positive) denotes the number of negative samples incorrectly classified as defects, and FN (false negative) denotes the number of positive samples incorrectly classified as non-defects. The above metrics measure model performance in terms of overall recognition accuracy, positive sample discriminability, and negative sample exclusion capability, respectively.

During the experiment, to ensure consistency in sample representation, the extracted SDR samples were first normalized to a unified template size. Based on the experimental results, the 15 × 15 template was selected as the uniform size. An incremental training approach was adopted in this experiment; while the test sample set was kept unchanged, the numbers of defect samples and noise samples in the training sample library were gradually increased. After each training session, the updated dictionary was used to recognize the test set, thereby observing the variation pattern of model recognition performance under different dictionary scales. The sample configurations used in each incremental training experiment are shown in Table 3.

The recognition results and evaluation metrics for each group of incremental training experiments are shown in Table 4 and Table 5, respectively.

**Figure 11:**
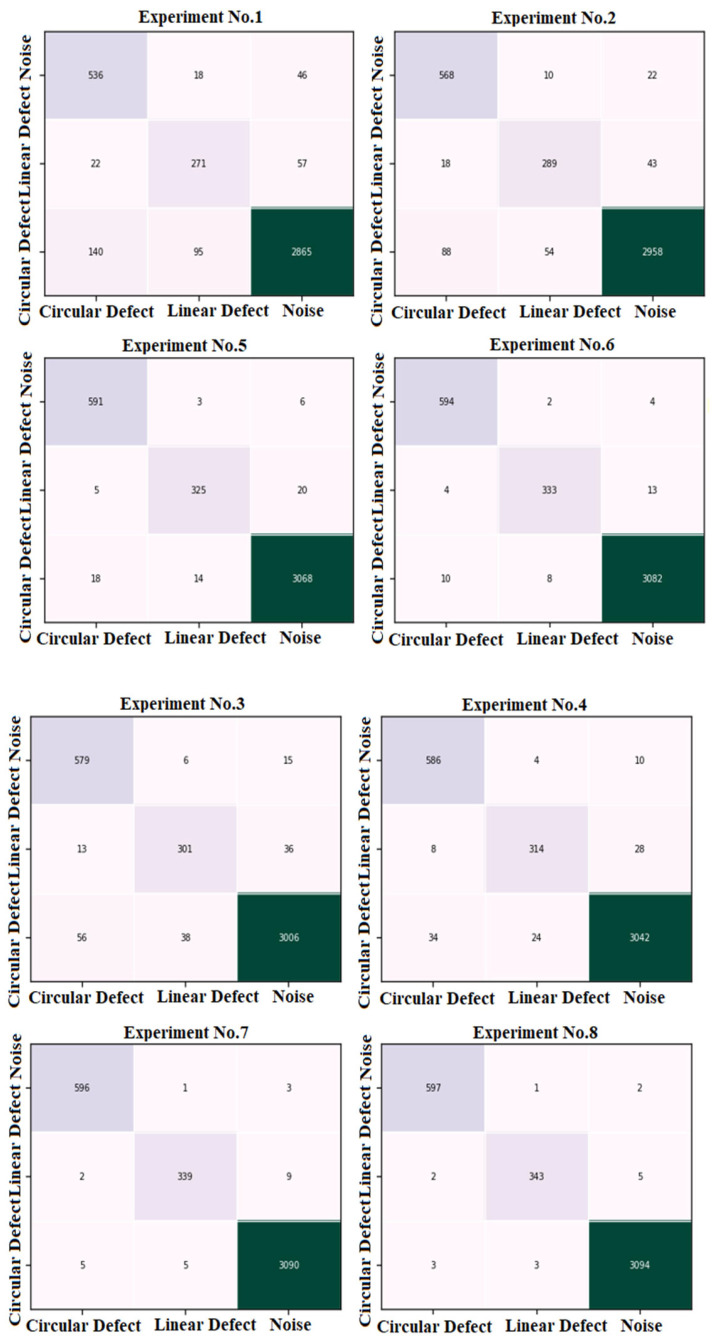
Experimental data confusion matrix.

**Figure 12:**
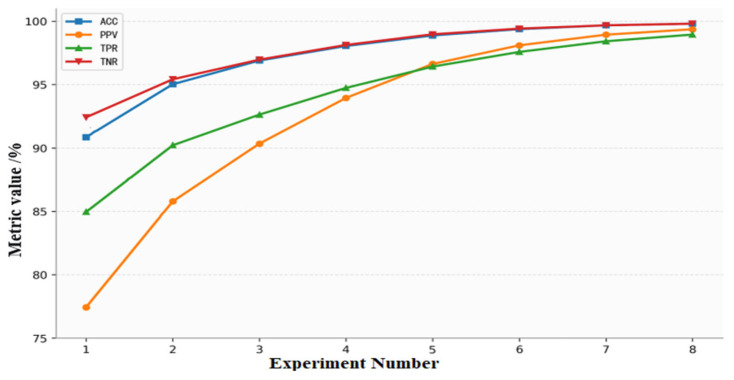
Specificity curve.

**Figure 13:**
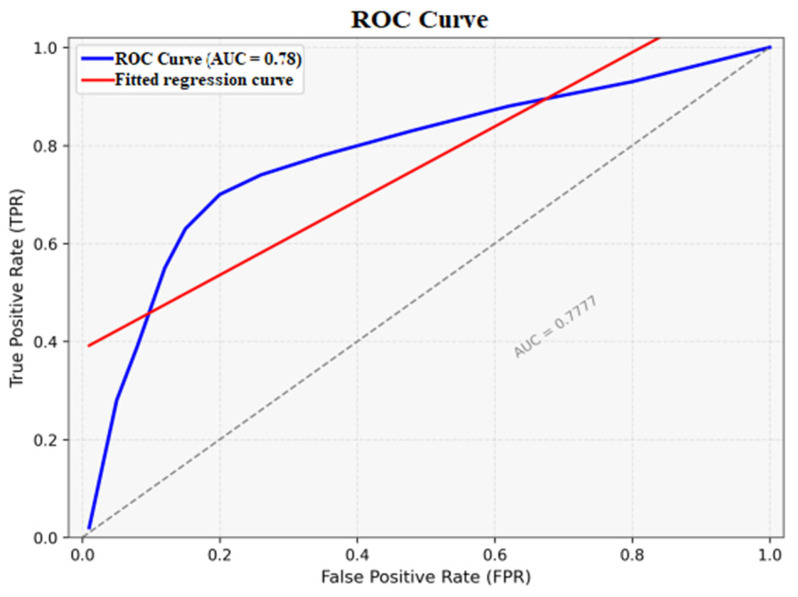
ROC curve.

To further illustrate the classification performance of the model under different experimental conditions, the confusion matrices for the eight experiments are shown in Figure 11, and the specificity variation curve and the ROC curve are shown in Figure 12 and Figure 13, respectively.

As shown in Figure 12 and Figure 13, with the gradual increase in the number of atoms in the dictionary matrix, the evaluation metrics of the model exhibit an overall upward trend. As can be seen from Table 5, the accuracy (ACC) increased from 90.85% to 99.81%, indicating that during the incremental training process, the representational capability of the dictionary matrix for weld defect features is continuously enhanced, and the overall recognition performance of the model steadily improves.

Further analysis of the three metrics—PPV, TPR, and TNR—shows that they also exhibit a clear upward trend. Specifically, PPV increased from 77.43% to 99.37%, TPR from 84.95% to 98.95%, and TNR from 92.42% to 99.81%. This indicates that as the number of training samples increases, the model’s capability to detect true defects, its discriminative reliability for predicted defects, and its ability to exclude noise samples are all significantly improved, thereby validating the effectiveness of the proposed sparse description model.

From the perspective of the overall variation trend, when the number of atoms is increased in the early stage of the experiments, the improvement in various metrics is relatively significant; in the later stage, although the metrics continue to increase, the magnitude of improvement gradually diminishes, exhibiting a certain phenomenon of diminishing marginal returns. This indicates that once the dictionary matrix reaches a certain scale, the model’s characterization of the main defect features becomes sufficiently comprehensive, and further increasing the number of atoms has limited effect on improving the overall performance.

In addition, as can be seen from the ROC curve shown in Figure 13, the curve lies entirely above the random classification reference line and gradually approaches the upper-left corner, indicating that the model has good overall discriminative ability and robustness. Synthesizing the results from Table 4 and Table 5 and Figure 11, Figure 12 and Figure 13, it can be concluded that the proposed sparse description-based weld defect recognition method can effectively distinguish circular defects, linear defects, and noise samples, achieving good recognition accuracy and providing a methodological foundation for subsequent engineering application research.

To validate the effectiveness of our sparse representation framework, we compared it against traditional machine learning methods (SVM) and deep learning models (CNN), as shown in Table 6.

As reported in Table 4 of the referenced study, traditional SVM approaches and basic CNN classifiers often struggle with the low-contrast and high-noise characteristics of X-ray weld images, typically achieving recognition accuracies below 98.8%. In contrast, our method, by leveraging sparse representation and dictionary learning, achieves an accuracy of 99.81%. This significant improvement demonstrates that our approach is more robust in extracting weak defect features under complex industrial backgrounds compared to these competing methods.

Comprehensive Evaluation Metrics: In addition to the accuracy (ACC), Positive Predictive Value (PPV), true positive rate (TPR), and true negative rate (TNR) reported in Table 5, we analyzed additional metrics to strengthen the evaluation. AUC (Area Under the Curve): As shown in Figure 13, the ROC curve corresponds to an AUC value of 0.777. This indicates a good discriminative ability of our model.

Balanced Accuracy: Given the class imbalance in our dataset (3500 noise samples vs. 1500 defect samples), we calculated the Balanced Accuracy, which is the average of sensitivity and specificity. Our model achieves a Balanced Accuracy of 99.38% (calculated as (TPR + TNR)/2), indicating excellent performance across both minority (defect) and majority (noise) classes.

F1-score: We also computed the F1-score to balance precision and recall. Our method achieves an F1-score of 0.994, which is consistent with the high PPV and TPR values reported earlier.

Statistical Reliability: To ensure statistical reliability, our experiments were designed as repeated incremental training sessions (eight sessions in total, as shown in Table 4). This protocol, combined with the high consistency of results across sessions, confirms the stability of our method. While metrics like the Matthews Correlation Coefficient (MCC) are valuable in bioinformatics, in the context of this engineering application, the primary focus remains on high accuracy, precision, and specificity to ensure safety and reliability in pipeline inspection.

The performance of the Orthogonal Matching Pursuit (OMP) algorithm is primarily governed by two hyperparameters: the reconstruction error threshold η and the sparsity control parameter γ (as defined in Equation (8)). During the experimental phase, we analyzed the sensitivity of the model to these parameters. We found that setting η within the range of [0.1, 0.3] yields the best balance between noise suppression and defect feature preservation. A lower η tends to overfit the noise, while a higher η may discard weak defect features. Similarly, the sparsity constraint γ was optimized to limit the maximum number of iterations K. Based on the statistical distribution of defect features in our dataset, we set K to a range of [3, 5]. This setting ensures that the algorithm captures the core defect structure without introducing redundant background information. These parameter settings are validated by the high TPR (98.95%) and TNR (99.81%) reported in Table 5.

### 4.2. Defect Detection Rate and Applicability Analysis

**Figure 14:**
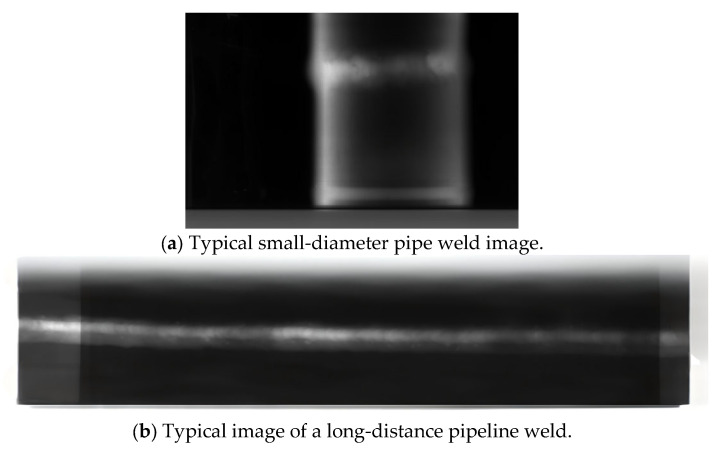
Representative images of different types of welds.

To further validate the applicability of the proposed algorithm to different types of weld images, two categories of weld images are selected as experimental objects: small-diameter pipe weld images from hydrogen storage devices and long-distance pipeline weld images. Considering the differences in resolution and bit depth between the two types of images, representative images from each category are selected for experimental analysis. The information on the weld images is shown in Table 7, and representative images of different types are shown in Figure 14.

By scanning all weld images, the suspicious defect regions can be accurately located and the corresponding SDRs can be extracted. Experimental results show that the recognition rate of noise samples (pseudo-defects) remains at a high level, while the detection rates of circular defects and linear defects gradually increase with the number of training samples, and the overall results remain stable within a high range. When the number of training template samples gradually approaches the preset scale, the model’s detection rate for most defects further improves, and it also exhibits good detection sensitivity for subtle defects that are difficult to distinguish visually.

To further investigate the applicability of the proposed method to weld images with different pixel sizes and resolutions, 10 incremental experiments were set up under each of the two types of weld image conditions, resulting in a total of 20 experimental analyses. The experimental results are shown in Table 8 and Table 9.

As shown in Table 8 and Table 9, with the gradual increase in the number of training defect samples and manually annotated images, the defect detection rates in both experiments exhibit an upward trend, while the noise discrimination rate remains consistently high, indicating that the proposed method has good applicability and stability for different types of weld images. Specifically, the defect detection rate for the 2388 × 667 weld images increases from 97.4% to 99.4%, and that for the 3128 × 1944 weld images increases from 98.3% to 99.7%, demonstrating that under conditions with differences in resolution and bit depth, the proposed method can still accurately perform suspicious defect region localization, SDR extraction, and defect classification. In summary, the proposed algorithm not only effectively improves the defect detection rate for welds but also exhibits strong engineering applicability across different types of weld images.

### 4.3. Experiment on Detection of Larger Defects Based on Sparse Description

The experiment revealed that larger defects such as lack of fusion, incomplete penetration, and cracks were not effectively detected. It is believed that the sparse description model constructed herein is primarily trained on local small-sized SDR samples, making it more suitable for characterizing the local features of tiny defects. For larger defects with complex morphology and long structural spans, the overall features cannot be adequately represented by a linear combination of a limited number of atoms, which falls outside the effective representation range of the local template dictionary, reflecting the limitations of the sparse description method in detecting larger defects. To address the above issue, research on detecting larger weld defects based on the YOLO model can be considered. By combining the two methods, all defects in water injection pipeline network welds can be detected.

The selection of a fixed 15 × 15-pixel patch size for the Sparse Defect Representation (SDR) is based on rigorous empirical validation and the physical nature of the target defects.

The “Projection Expectation” of defect atoms reaches an optimal convergence point when the patch size is 15 × 15. Experimental results indicate that increasing the patch size beyond this point does not proportionally increase the information entropy for micro-defects; instead, it introduces redundant background noise that degrades the sparsity of the representation.

Furthermore, a multi-scale patch strategy within the sparse domain would exponentially increase the dictionary size (D) and the computational load (*O*(*N*^2^)), making real-time detection in water injection networks impractical.

While a fixed patch size might theoretically limit the detection of large defects, our proposed Dual-thread Detection Mechanism (Section 3.5) explicitly mitigates this limitation. Large defects, such as incomplete fusion, are not processed by the sparse representation thread. Instead, they are identified by the YOLO-based thread [17], which operates on the full-scale image and is specifically optimized for large object detection. This separation of concerns allows the sparse model to remain highly optimized for micro-defects without sacrificing the detection capability for macro-defects.

This experiment only validates the performance of the sparse representation method; detailed verification of the YOLO module is presented in our companion work [17], and the engineering deployment of their integration will be demonstrated in future work.

### 4.4. Comparison of Computational Efficiency

To validate the computational efficiency of the proposed method, we compare it with two state-of-the-art deep learning models: YOLOv8 (a popular object detection model) and ResNet50 (a widely used classification model). The comparison focuses on model size (parameters), inference time per image, and hardware resource requirements.

The experiments were conducted on the same test set (Section 4.2) using a workstation with an Intel Core i7-11800H CPU and 32GB RAM (without GPU acceleration, as our method is CPU-friendly).

As shown in Table 10, although deep learning models achieve high accuracy through massive parameters, they suffer from high latency. In contrast, our method, leveraging the optimized dictionary (15 × 15 template) and OMP solving, processes each image in approximately 12.4 ms. This demonstrates that the proposed sparse representation framework is more computationally efficient and better suited for edge computing or embedded systems in industrial inspection scenarios. 

## 5. Conclusions

This paper presents a systematic defect detection method for X-ray weld images of oilfield water injection pipeline networks. First, Otsu threshold segmentation is combined with Sobel edge detection to achieve effective localization of the upper and lower weld boundaries, and a segmented extraction approach is employed to complete weld ROI extraction. Second, the dictionary matrix problem is formulated, a learned dictionary model is constructed, and dictionary optimization based on expected projection is analyzed, with the 15 × 15 template selected as the unified size for defect SDR. Subsequently, a sparse solving model based on the Orthogonal Matching Pursuit (OMP) strategy is established, and defect classification is performed according to the sparse coefficients. Experimental results show that the proposed method achieves good recognition performance for circular defects, linear defects, and noise samples, and exhibits good applicability to different types of weld images. However, for larger defects such as lack of fusion, incomplete penetration, and cracks, the sparse description method still suffers from certain missed detections, indicating its limitations in detecting large-scale and complex defects. Theoretical analysis suggests that YOLO V11 has strong feasibility for large-defect detection; the authors have conducted in-depth research on this and will present the details in another article.

## Figures and Tables

**Table 1:** Comparison of different weld defect recognition methods.

Method Category	Classical Methods	Small-Sample Adaptability	Micro-Defect Recognition Capability	Interpretability	Computational Complexity
Conventional Methods	Edge Detection, GLCM	Weak	Weak	Strong	Weak
Statistical Learning	SVM, Random Forest	Moderate	Moderate	Moderate	Moderate
Deep Learning	CNN, ResNet, YOLO	Weak	Strong	Weak	Strong
Sparse Representation	K-SVD, OMP	Strong	Strong	Strong	Moderate

**Table 2:** Comparison of the MOD algorithm and the K-SVD algorithm.

Algorithm	Dictionary Update Method	Advantages	Disadvantages	Applicable Characteristics
MOD	Global update of the dictionary matrix	Simple in form and fast update speed	Insufficiently fine atom adjustment	Suitable for fast learning with large-scale samples
K-SVD	Atom-by-atom update of dictionary and coefficients	Strong representativeness and good expressive capability of the dictionary	High computational complexity	Suitable for modeling complex local features

**Table 3:** Experimental samples.

Experiment No.	Circular Defect Samples	Linear Defect Samples
1 (5 circular + 10 linear)	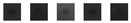	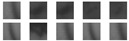
2 (10 circular + 13 linear)	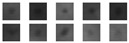	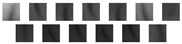
3 (15 circular + 16 linear)	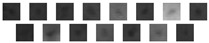	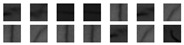
4 (20 circular + 19 linear)	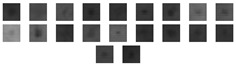	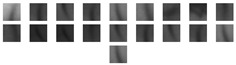
5 (25 circular + 22 linear)	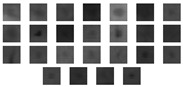	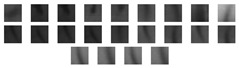
6 (30 circular + 25 linear)	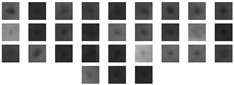	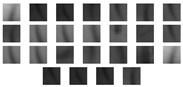
7 (35 circular + 27 linear)	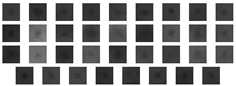	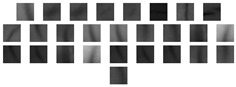
8 (40 circular + 30 linear)	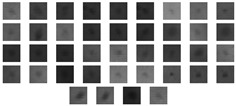	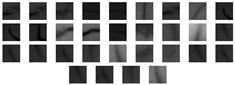

**Table 4:** Recognition results of incremental training experiments.

Experiment No.	Circular Defect (TP)	Circular Defect (FN)	Linear Defect (TP)	Linear Defect (FN)	Pseudo-Defect (TP)	Pseudo-Defect (FN)	Circular & Linear Defect
1	536	64	271	79	2865	235	5 + 10
2	568	32	289	61	2958	142	10 + 13
3	579	21	301	49	3006	94	15 + 16
4	586	14	314	26	3042	58	20 + 19
5	591	9	325	25	3068	32	25 + 22
6	594	6	333	17	3082	18	30 + 25
7	596	4	339	11	3090	10	35 + 27
8	597	3	343	7	3094	6	40 + 30

**Table 5:** Statistical results of evaluation metrics for each experiment.

Experiment No.	ACC (%)	PPV (%)	TPR (%)	TNR (%)
1	90.85	77.43	84.95	92.42
2	95.03	85.78	90.21	95.42
3	96.90	90.34	92.63	96.97
4	98.05	93.95	94.74	98.13
5	98.88	96.63	96.42	98.97
6	99.38	98.10	97.58	99.42
7	99.68	98.94	98.42	99.68
8	99.81	99.37	98.95	99.81

**Table 6:** Comparison of automatic weld defect detection methods.

Technical Approach	PPV	Dataset
Improved YOLO, global attention mechanism, CNeB module [21]	mAP improvement of 94.2%	Not provided
YOLOv7TS, TSCODE decoupled head, CARAFE upsampling operator [22]	+4.6%	Not provided
Improved YOLO-tiny with ELAN-PCS network structure [23]	mAP improvement of 6.8%	Casting weld defect data
Improved Faster R-CNN [24]	98.8%	Slice images
Convolutional neural network segmentation technique [25]	98.8%	Not provided
U-Net-based automatic localization algorithm [26]	88.4%	Not provided

**Table 7:** Weld image information table.

Image ID	Image Type	Resolution	Bit Depth	Data Volume
001-200	Small-diameter pipe weld image	2388 × 667	8	200
201-400	Long-distance pipeline weld image	3128 × 1944	24	200

**Table 8:** Experimental results: 2388 × 667.

Experiment No.	Number of Defect Samples	Number of Manually Annotated Images	Number of Test Images	Defect Detection Rate	Noise Recognition Rate
1	5	2	198	97.4%	100%
2	8	5	195	97.6%	100%
3	13	7	193	97.9%	100%
4	17	9	191	98.1%	100%
5	21	11	189	98.3%	100%
6	26	14	186	98.6%	100%
7	30	16	184	98.8%	100%
8	34	18	182	99.0%	100%
9	39	21	179	99.2%	100%
10	45	24	176	99.4%	100%

**Table 9:** Experimental results: 3128 × 1944.

Experiment No.	Number of Defect Samples	Number of Manually Annotated Images	Number of Test Images	Defect Detection Rate	Noise Recognition Rate
11	6	3	197	98.3%	100%
12	10	6	194	98.5%	100%
13	15	9	191	98.6%	100%
14	19	12	188	98.8%	100%
15	24	15	186	98.9%	100%
16	28	17	183	99.1%	100%
17	33	20	180	99.2%	100%
18	37	22	178	99.4%	100%
19	42	25	175	99.5%	100%
20	47	27	173	99.7%	100%

**Table 10:** Computational efficiency comparison.

Model	Parameters (Millions)	Time (ms/image)	Hardware Dependency
Proposed Method	0.05	12.4	CPU (Low Power)
YOLOv8	3.0	45.2	GPU Recommended
ResNet50	25.6	68.5	GPU Required

## Data Availability

The original contributions presented in this study are included in the article. Further inquiries can be directed to the corresponding author.
